# Supplementary material for: Cytotoxic, Anti-Hemolytic, and Antioxidant Activities of Ruta chalepensis L. (Rutaceae) Extract, Fractions, and Isolated Compounds
Source: Plants (Basel). 2023 Jun 2;12(11):2203. doi: 10.3390/plants12112203 (PMC10255231; doi:10.3390/plants12112203)
Supplement: Supplementary file 1 [file plants-12-02203-s001.zip › Supplementary Material_Herbarium Identification Voucher.pdf]

UANL
UNIVERSIDAD AUTÓNOMA DE NUEVO LEÓN

FACULTAD DE CIENCIAS BIOLÓGICAS
DEPARTAMENTO DE BOTÁNICA
HERBARIO UNL

A QUIEN CORRESPONDA:

Por este conducto me permito informar que el Dr. RAMIRO QUINTANILLA LICEA entregó al Herbario UNL dieciséis (16) muestras herbolarias, como parte del Proyecto Académico: Capacidad antitumoral, citotóxica y citoprotectora *in vitro* e *in vivo* de moléculas presentes en plantas de origen mexicano de uso etnofarmacológico tradicional, del cual es Asesor.
La referencia de las muestras entregadas se presenta en la siguiente tabla e incluye el número de registro en la colección de nuestro herbario institucional.

MATERIAL VEGETAL

No. de Folio	Género	Especie	Familia
30642	*Amphipterygium*	*adstringens*	Anacardiaceae
30643	*Artemisia*	*ludoviciana*	Asteraceae
30644	*Cymbopogon*	*citratus*	Poaceae
30645	*Haematoxylum*	*brasiletto*	Fabaceae
30646	*Heterotheca*	*inuloides*	Asteraceae
30647	*Hippocratea*	*excelsa*	Celastraceae
30648	*Jatropha*	*dioica*	Euphorbiaceae
30649	*Justicia*	*spicigera*	Acanthaceae
30650	*Larrea*	*tridentata*	Zygophyllaceae
30651	*Mimosa*	*tenuiflora*	Fabaceae
30652	*Psacalium*	*peltatum*	Asteraceae
30653	*Pseudognaphalium*	*obtusifolium*	Asteraceae
30654	*Ruta*	*chalepensis*	Rutaceae
30655	*Smilax*	*aspera*	Smilacaceae
30656	*Tagetes*	*lucida*	Asteraceae
30657	*Turnera*	*diffusa*	Turneraceae

Sin más por el momento, se extiende la presente para los fines que al interesado convengan.

ATENTAMENTE
Cd. Universitaria, a 7 de Noviembre de 2021

Dr. Marco Antonio Guzmán Lucio
Jefe y Curador del Herbario UNL

c.c.p. archivo, Herbario UNL

Pedro de Alba y Manuel Barragán s/n
Cd. Universitaria, San Nicolás de los Garza, N. L.
Apartado Postal F-2 y F-38, C.P. 66451
Tels y Fax. 8329-4110 ext. 6456, 8298-2126 y 8114-3465
