# Supplementary material for: Cytotoxic, Anti-Hemolytic, and Antioxidant Activities of Ruta chalepensis L. (Rutaceae) Extract, Fractions, and Isolated Compounds
Source: Plants (Basel). 2023 Jun 2;12(11):2203. doi: 10.3390/plants12112203 (PMC10255231; doi:10.3390/plants12112203)
Supplement: Supplementary file 1 [file plants-12-02203-s001.zip › Supplementary Material_Institutional Review Board approval CI-04-26-2022.pdf]

UNIVERSIDAD AUTÓNOMA DE NUEVO LEÓN FACULTAD DE CIENCIAS BIOLÓGICAS

SUBJECT: Research project evaluation

Dr. Ricardo Gómez Flores:

By this means, you are informed that in a session held on April 26, 2022, the research project with registration number CI-04-26-2022 “Antitumor activity of methanolic extracts of Mexican plants on tumor cell lines” was reviewed and approved.

The global scientific evaluation of this research project complies with technical-scientific quality, scientific relevance and impact, as well as with the criteria, guidelines and institutional requirements.

Sincerely yours,

Atentamente
“*ALERE FLAMMAM VERITATIS*”
Cd. Universitaria a 26 de abril del 2022

COMITÉ DE INVESTIGACIÓN

Dr. Juan Francisco Contreras
Presidente

Dra. María Julissa Ek Ramos
Secretario

Pedro de Alba y Manuel L. Barragán s/n, Cd. Universitaria, San Nicolás de los Garza, Nuevo León, México.
Tel. (81) 83294110 ext. 6432
