# Supplementary material for: Cytotoxic, Anti-Hemolytic, and Antioxidant Activities of Ruta chalepensis L. (Rutaceae) Extract, Fractions, and Isolated Compounds
Source: Plants (Basel). 2023 Jun 2;12(11):2203. doi: 10.3390/plants12112203 (PMC10255231; doi:10.3390/plants12112203)
Supplement: Supplementary file 1 [file plants-12-02203-s001.zip › Supplementary Material_Purification Methodology.pdf]

*plants*

MDPI

*Article*

# Cytotoxic, Anti-hemolytic, and Antioxidant Activities of *Ruta chalepensis* L. (Rutaceae) Extract, Fractions, and Isolated Compounds

**Supplementary Material**

**Summary**

This research reported the *in vitro* cytotoxic activity of *Ruta chalepensis* methanol (MeOH) extract and *n*-hexane, chloroform ($CHCl_3$), and MeOH sub-partitions. In addition, we demonstrated the cytotoxic activity of previously isolated *R. chalepensis* bioactive compounds chalepensin (CHL), rutamarin (RTM), and graveoline (GRV). We reported the isolation of CHL in DOI: 10.3390/molecules191221044 [1], RTM in DOI: 10.3390/molecules26123684 [2], and GRV in DOI: 10.1055/s-0036-1596528 [3], whose identification was based on spectroscopic/spectrometric analysis obtained by a Bruker Spectrometer (Model Advance DPX400, 9.4 Teslas; Bruker Corporation, Billerica, MA, USA) and in comparison with bibliographic data. All structures were matched on the PubChem website (https://pubchem.ncbi.nlm.nih.gov/). The spectroscopic analysis data of CHL, RTM, and GRV are available as supplementary material (Supplementary Material_Spectroscopic Data).

**Methods**

Steps for purification are explained as follows: *R. chalepensis* crude MeOH extract was dissolved in 200 mL of MeOH, after which 600 mL of *n*-hexane was added to obtain a liquid-liquid partition. The *n*-hexane partition was vacuum evaporated. Its column chromatography on silica gel resulted in the isolation of 243 mg of CHL (colorless needles; melt point (MP) = 75 °C; $R_f$ = 0.41 in *n*-hexane–$CHCl_3$) with a molecular formula of $C_{16}H_{14}O_3$ (6-(2-methylbut-3-en-2-yl)furo[3,2-g]chromen-7-one) and a molecular weight (MW) of 254.28 [1].

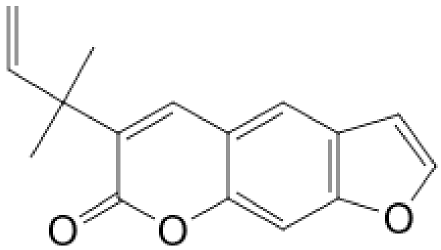

**Figure S1.** Structure of chalepensin (CHL). PubChem CID = 128834 (https://pubchem.ncbi.nlm.nih.gov/compound/Chalepensin).

MeOH phase was vacuum concentrated until obtaining 50 mL, which was added dropwise into 150 mL of distilled $H_2O$ under continuous agitation. Resulting suspension

was processed to a liquid–liquid partition with EtOAc (600 mL). After EtOAc evaporation (20.0 g of resin was obtained), the partition was subjected to open column chromatography (30 cm × 2 cm) on 25 g of silica gel, eluting with stepwise 50 mL of $CHCl_3$-EtOAc gradients (100:0, 90:10, 80:20, 70:30, 60:40, 50:50, 40:60, 30:70, 20:80, 10:90, and 0:100 v/v) and 50 mL of EtOAc-MeOH (100:0, 90:10, 80:20, 70:30, 60:40, and 50:50 v/v). Obtained fractions were pooled based on their TLC ($CHCl_3$-EtOAc; 9.5:0.5) profile to yield nine fractions (A1 to A9). Fraction A3 (1.55 g) was subjected to open-column chromatography on silica gel and eluted with 50 mL of $CHCl_3$-EtOAc gradients (100:0, 90:10, 80:20, 70:30, 60:40, 50:50, and 40.60 v/v. Fractions were then pooled based on their TLC ($CHCl_3$-EtOAc; 9.5:0.5) profile in five fractions (B1 to B5). Fraction B2 (716 mg) was subjected to open-column chromatography on silica gel and eluted with 50 mL of $CHCl_3$-EtOAc gradients (100:0, 90:10, 80:20, 70:30, 60:40, 50:50, 40.60, 30:70, 20:80, 10:90, and 0:100 v/v). Obtained fractions were pooled according to their TLC (CHCl3-EtOAc; 9.5:0.5) profile in four fractions (C1 to C4). Fraction C2 (540 mg) was subjected to open-column chromatography on silica gel and eluted with 50 mL of $CHCl_3$-EtOAc gradients (100:0, 99:01, 95:05, 90:10, 85:15, 80:20, 70:30, and 60:40 v/v). Fractions obtained were pooled according to their TLC ($CHCl_3$-EtOAc; 9.5:0.5) profile in three fractions (D1 to D3). Fraction D2 (499 mg) was subjected to crystallization in MeOH, obtaining a white amorphous solid (365 mg) that was subjected to open column chromatography on Sephadex® LH-20 and eluted with MeOH. Obtained fractions were pooled according to their TLC ($CHCl_3$-EtOAc; 9.5:0.5) profile in five fractions (E1 to E5). Fraction E2 consisted of 252 mg of pure RTM with a molecular formula of $C_{21}H_{24}O_5$ (2-[6-(2-methylbut-3-en-2-yl)-7-oxo-2,3-dihydrofuro[3,2-g]chromen-2-yl]propan-2-yl acetate; MW = 356.41), $R_f$ = 0.66 ($CHCl_3$:EtOAc, 9.5:0.5) [2].

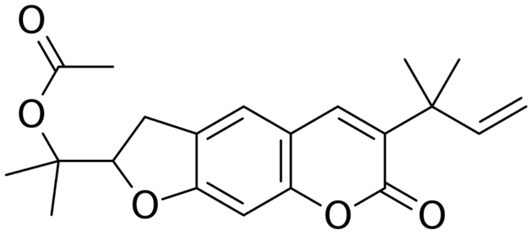

**Figure S2.** Structure of rutamarin (RTM). PubChem CID = 26948 (https://pubchem.ncbi.nlm.nih.gov/compound/26948).

To purify GRV, fractions A6, A7, and A8 were obtained (1.5 g) and subjected to open-column chromatography on silica gel (20 g) and eluted with 50 mL $CHCl_3$-EtOAc gradients (100:0, 90:10, 80:20, 70:30, 60:40, 50:50, 40.60, 30:70, 20:80, 10:90, and 0:100 v/v) and 50 mL of MeOH. Obtained fractions were pooled according to their TLC ($CHCl_3$-EtOAc; 9:1) profile in six fractions (F1 to F6). Fraction F5 (336 mg) was subjected to open-column chromatography on reversed-phase silica gel and eluted with MeOH. Obtained fractions were pooled according to their TLC ($CHCl_3$-EtOAc; 1:1) profile in three fractions (G1 to G3). Fraction G2 (232 mg) was subjected to open-column chromatography on Sephadex® LH-20 and eluted whit 450 mL of MeOH. Obtained fractions were pooled according to their TLC ($CHCl_3$-EtOAc; 1:1) profile in four fractions (H1 to H4). Fraction H3 (142 mg) was subjected to open-column chromatography on Sephadex® LH-20 and eluted with 300 mL of MeOH. Obtained fractions were pooled according to their TLC ($CHCl_3$-EtOAc; 1:1) profile in two fractions (I1 and I2). Fraction I2 resulted in 17 mg of pure GRV (colorless needles; MP 167.82 °C; $R_f$ = 0.23 in CHCl3-EtoAc; 1:1). Considering all the information provided by the NMR techniques, the structure of the compound was confirmed as 2-(1,3-

benzodioxol-5-yl)-1-methylquinolin-4-one, commonly known as graveolin (GRV), with a molecular formula of $C_{17}H_{13}NO_3$ (MW = 279.29) [3].

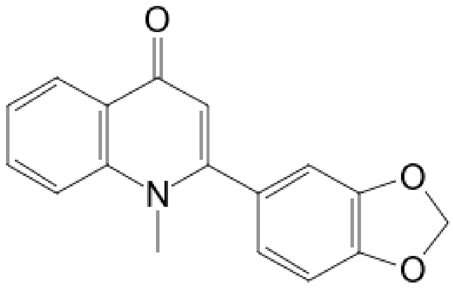


**Figure S3.** Structure of graveoline (GRV). PubChem CID = 353825 (https://pubchem.ncbi.nlm.nih.gov/compound/353825).

**Data Availability Statement:** The datasets generated or analyzed during the present study are available from the corresponding authors.

**Conflicts of Interest:** The authors declare no conflict of interest.

## References

1. Quintanilla-Licea, R.; Mata-Cárdenas, B.D.; Vargas-Villarreal, J.; Bazaldúa-Rodríguez, A.F.; Ángeles-Hernández, I.K.; Garza-González, J.N.; Hernández-García, M.E. Antiprotozoal activity against *Entamoeba histolytica* of plants used in northeast mexican traditional medicine. Bioactive compounds from *Lippia graveolens* and *Ruta chalepensis*. *Molecules* **2014**, *19*, 21044–21065, doi:10.3390/molecules191221044.
2. Bazaldúa-Rodríguez, A.F.; Quintanilla-Licea, R.; Verde-Star, M.J.; Hernández-García, M.E.; Vargas-Villarreal, J.; Garza-González, J.N. Furanocoumarins from *Ruta chalepensis* with amebicide activity. *Molecules* **2021**, *26*, 1–11, doi:10.3390/molecules26123684.
3. Quintanilla-Licea, R.; Mata-Cárdenas, B.; Vargas-Villarreal, J.; Bazaldúa-Rodríguez, A.; Verde-Star, M. Antiprotozoal activity against *Entamoeba histolytica* of furocoumarins isolated from *Ruta chalepensis*. *Planta Med.* **2016**, *81*, S1–S381, doi:10.1055/s-0036-1596528.
