# Supplementary material for: Cytotoxic, Anti-Hemolytic, and Antioxidant Activities of Ruta chalepensis L. (Rutaceae) Extract, Fractions, and Isolated Compounds
Source: Plants (Basel). 2023 Jun 2;12(11):2203. doi: 10.3390/plants12112203 (PMC10255231; doi:10.3390/plants12112203)
Supplement: Supplementary file 1 [file plants-12-02203-s001.zip › Supplementary Material_Spectroscopic Data.pdf]

plants

MDPI

Article

Cytotoxic, Anti-hemolytic, and Antioxidant Activities of *Ruta chalepensis* L. (Rutaceae) Extract, Fractions, and Isolated Compounds

Spectroscopic Data

Summary

This research reported the previous isolation of chalepensin (CHL; Figure 1.1) in DOI: 10.3390/molecules191221044 [1], rutamarin (RTM; Figure 2.1) in DOI: 10.3390/molecules26123684 [2], and graveoline (GRV; Figure 3.1) in DOI: 10.1055/s-0036-1596528 [3], whose identification was based on spectroscopic/spectrometric analysis obtained by a Bruker Spectrometer (Model Advance DPX400, 9.4 Teslas; Bruker Corporation, Billerica, MA, USA) and in comparison with bibliographic data; all structures were matched on the PubChem website (https://pubchem.ncbi.nlm.nih.gov/).

Spectroscopic Data

Chalepensin (CHL)

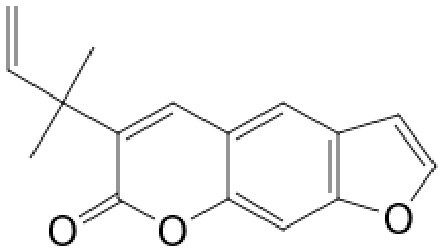

Figure S1.1. Structure of chalepensin (CHL). PubChem CID = 128834 (https://pubchem.ncbi.nlm.nih.gov/compound/Chalepensin).

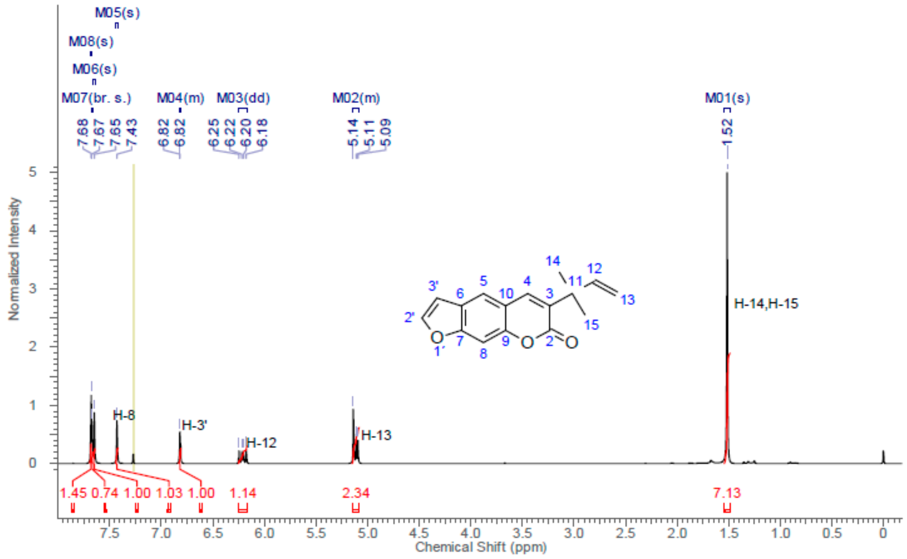

Figure S1.2.1. ^{1}H-NMR spectrum of CHL (Deuterochloroform (CDCl$_3$), 400 MHz).

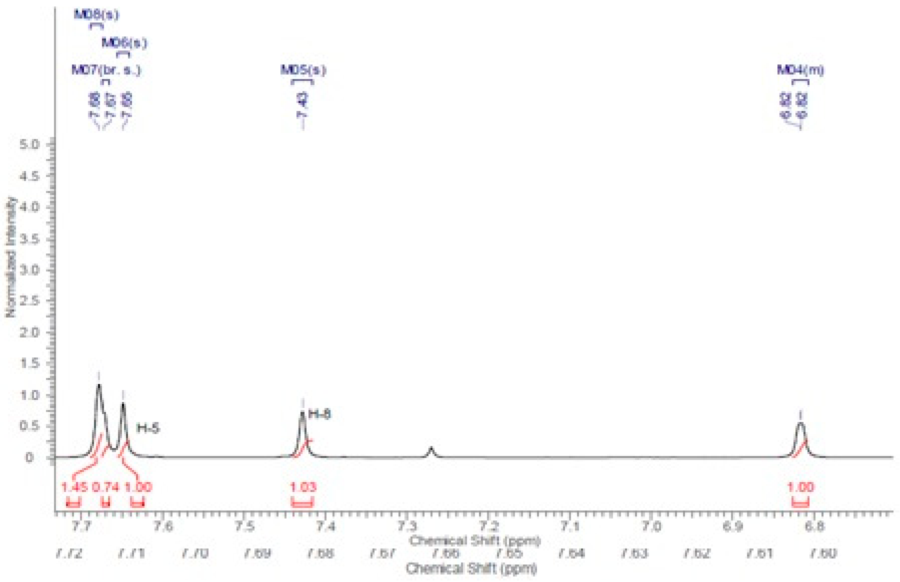

Figure S1.2.2. ^{1}H-NMR spectrum of CHL ($CDCl_3$, 400 MHz). Expansion in the area from 7.7 - 6.7 ppm.

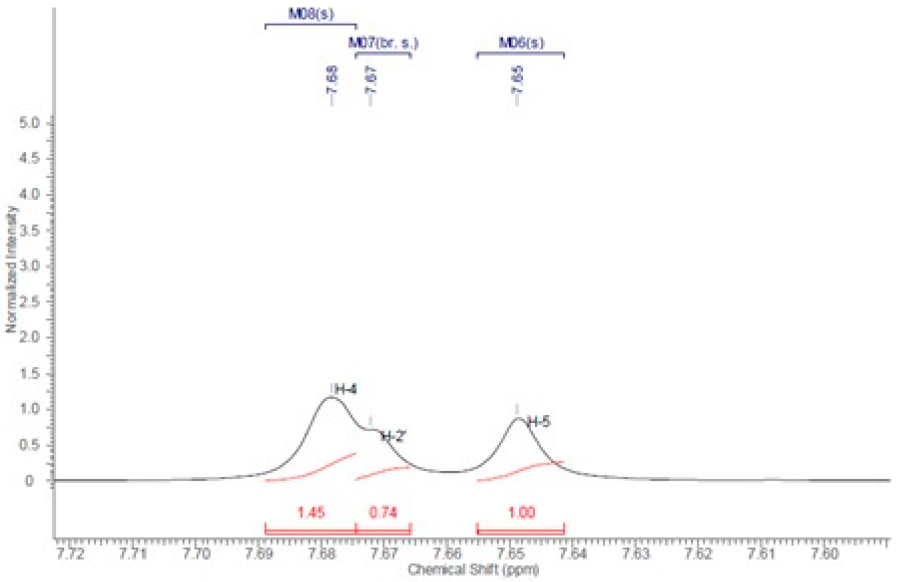

Figure S1.2.3. ^{1}H-NMR spectrum of CHL ($CDCl_3$, 400 MHz). Expansion in the area from 7.72 – 7.60 ppm.

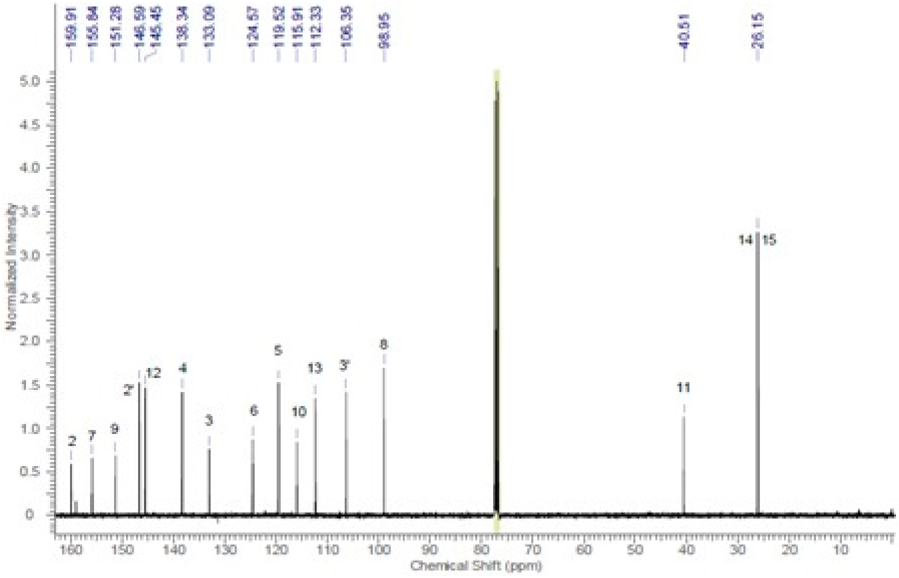

Figure S1.3. ^{13}C-NMR spectrum of CHL ($CDCl_3$, 400 MHz).

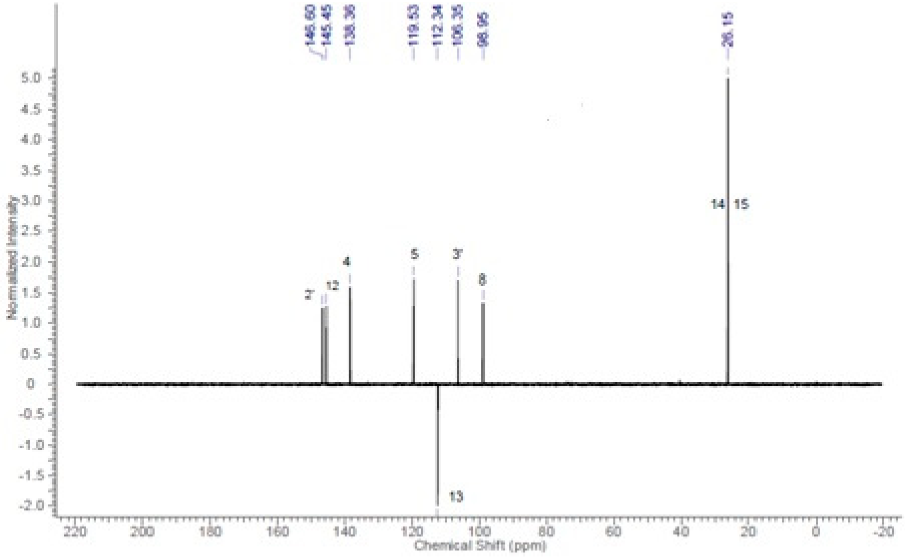

Figure S1.4. DEPT-135 spectrum of CHL ($CDCl_3$, 100 MHz).

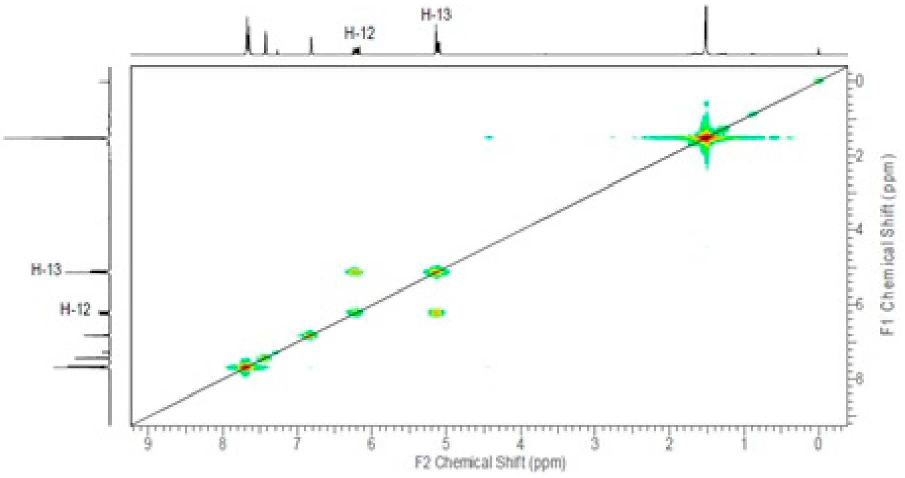

Figure S1.5. ^{1}H-^{1}H COSY spectrum of CHL ($CDCl_3$, 400 MHz).

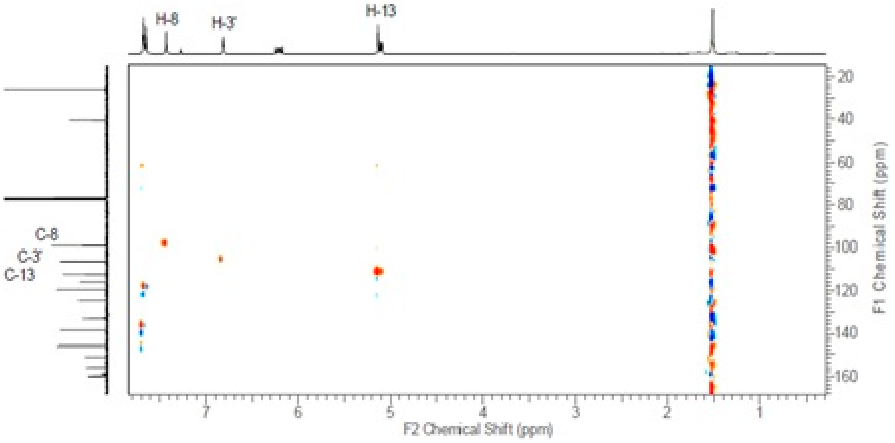

Figure S1.6.1. ^{1}H-^{13}C-HSQC spectrum of CHL ($CDCl_3$, 100 MHz).

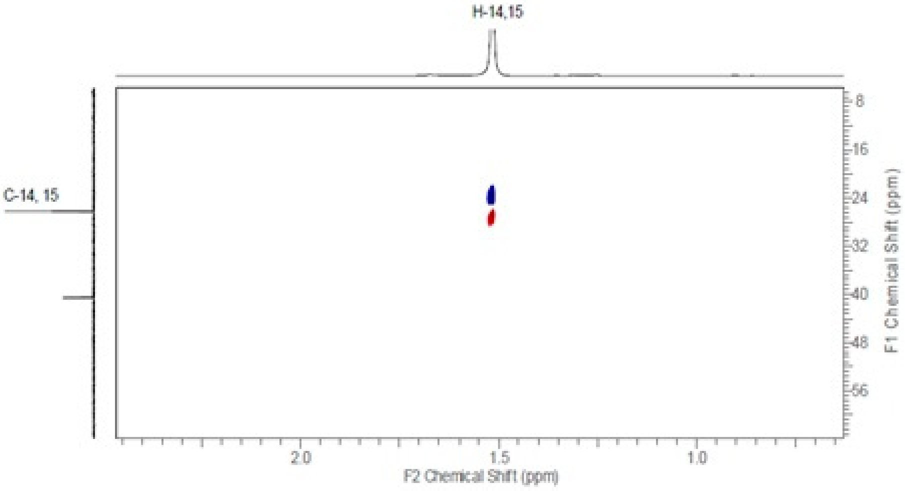

Figure S1.6.2. ^{1}H-^{13}C-HSQC spectrum of CHL ($CDCl_3$, 100 MHz). Expansion of the area ^{1}H: 0.5 - 2.7 ppm, ^{13}C: 6.0 - 64 ppm.

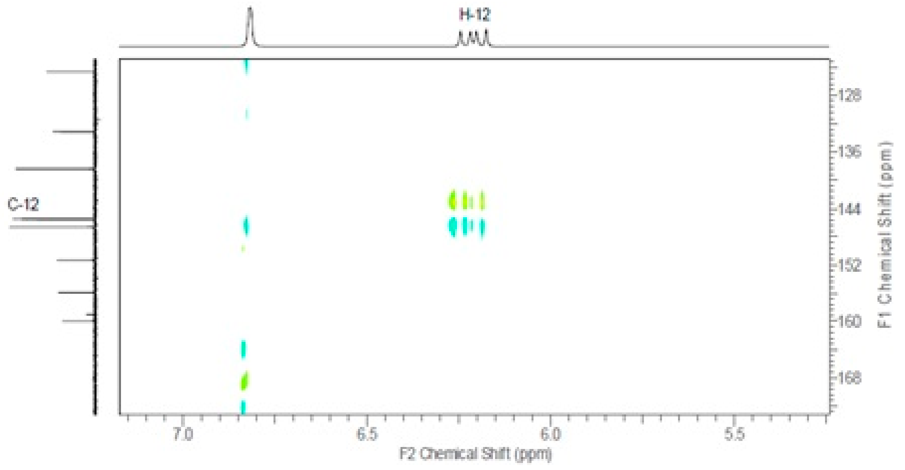

Figure S1.6.3. ^{1}H-^{13}C-HSQC spectrum of CHL ($CDCl_3$, 100 MHz). Expansion of the area ^{1}H: 5.3 - 7.1 ppm, ^{13}C: 123 - 172 ppm.

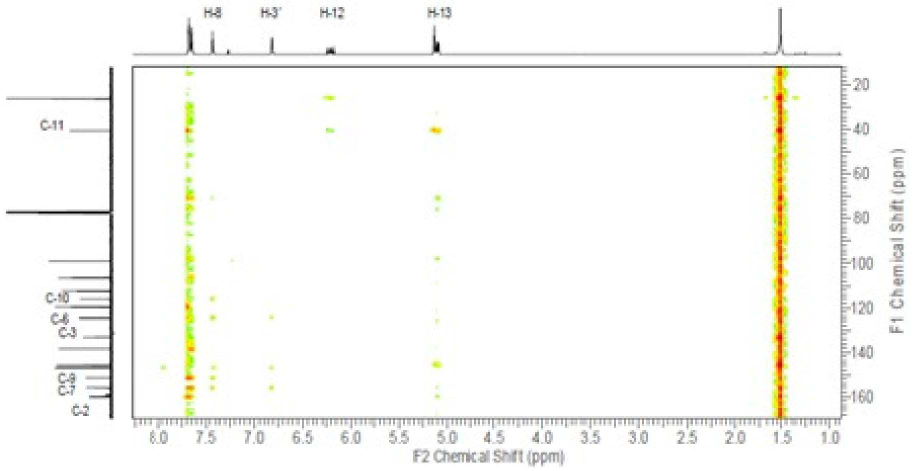

Figure S1.7. ^{1}H-^{13}C-HMBC spectrum of CHL ($CDCl_3$, 100 MHz).

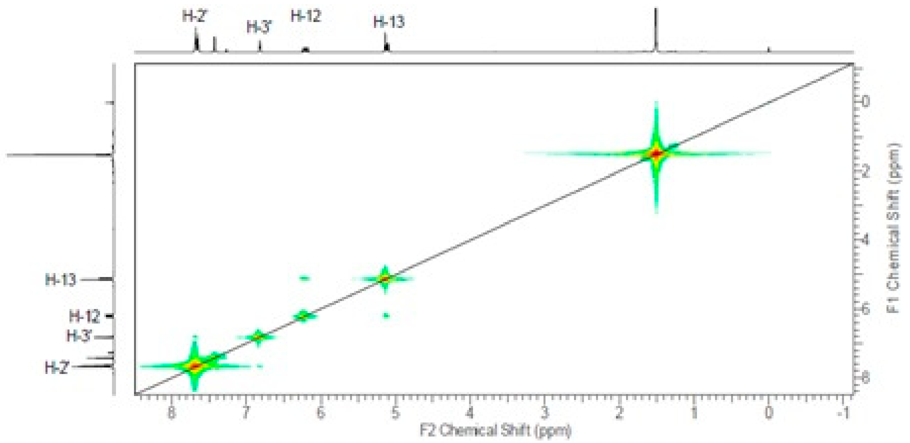

Figure S1.8. ^{1}H-^{1}H-NOESY spectrum of CHL ($CDCl_3$, 400 MHz).

Rutamarin (RTM)

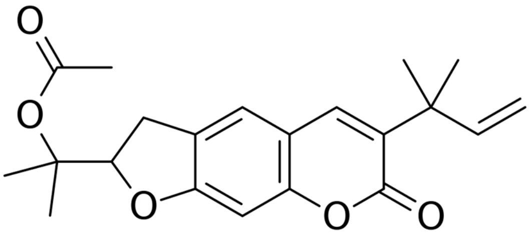

Figure S2.1. Structure of rutamarin (RTM). PubChem CID = 26948 (https://pubchem.ncbi.nlm.nih.gov/compound/26948).

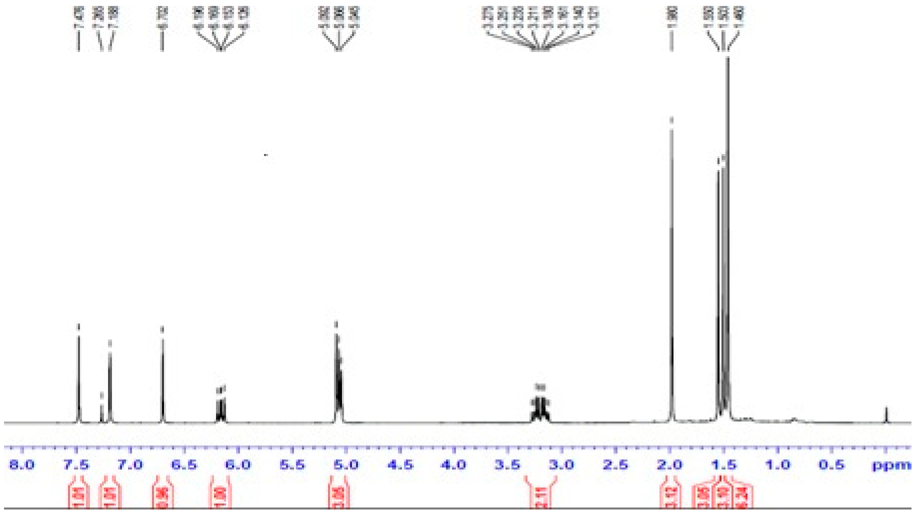

Figure S2.2.1. ^{1}H-NMR spectrum of RTM ($CDCl_3$, 400 MHz).

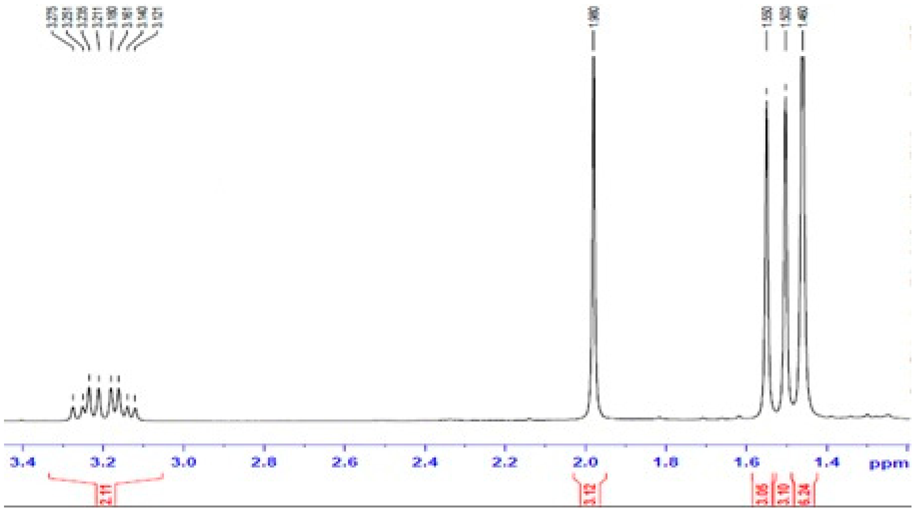

Figure S2.2.2. ^{1}H-NMR spectrum of RTM ($CDCl_3$, 400 MHz). Expansion in the area from 1.2 - 3.4 ppm.

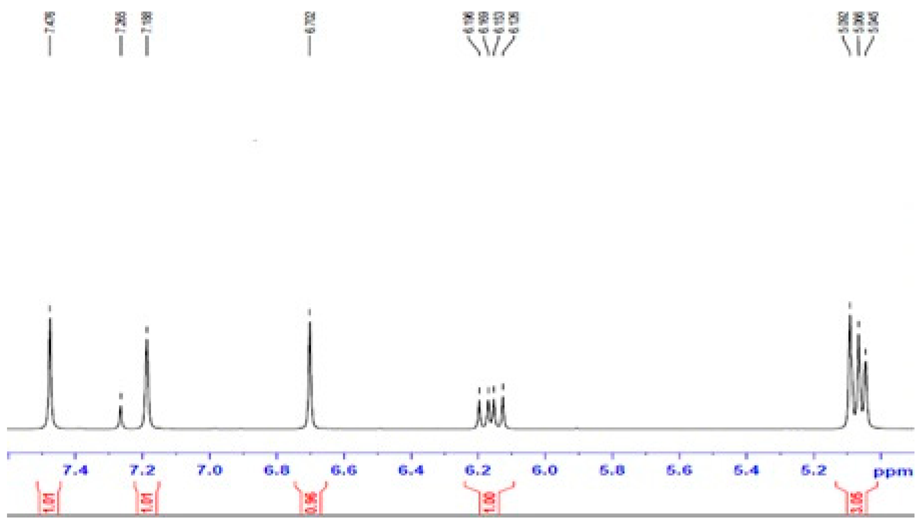

Figure S2.2.3. ^{1}H-NMR spectrum of RTM ($CDCl_3$, 400 MHz). Expansion in the area from 4.8 - 7.6 ppm.

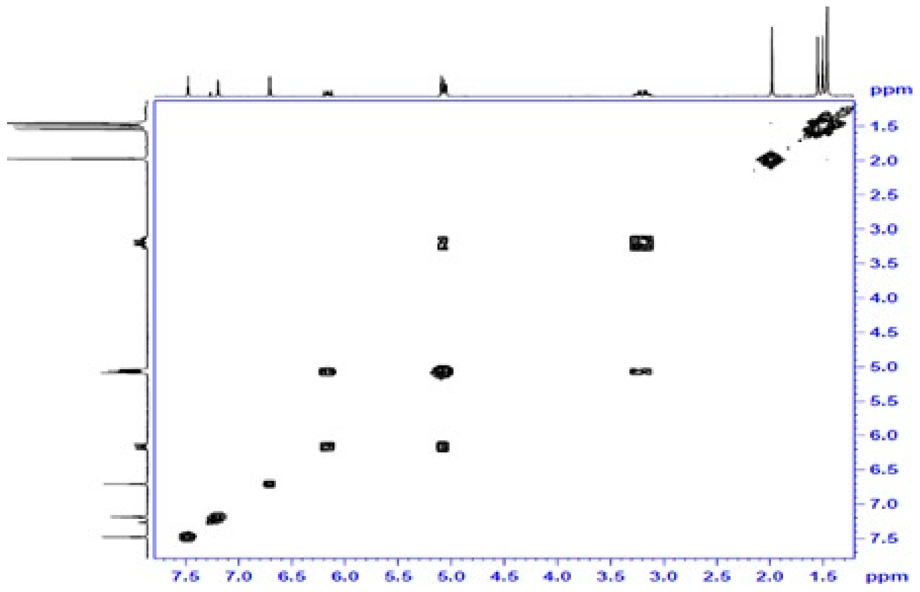

Figure S2.3. ^{1}H-^{1}H COSY spectrum of RTM ($CDCl_3$, 400 MHz).

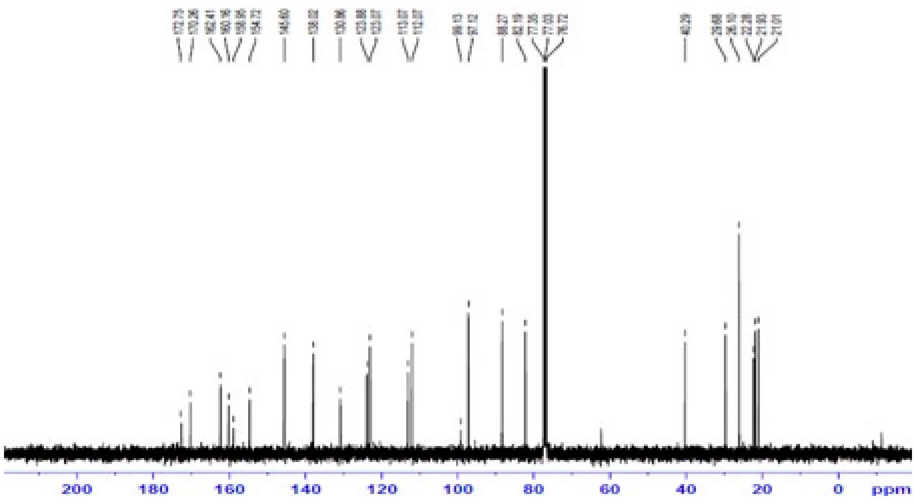

Figure S2.4.1. ^{13}C-NMR spectrum of RTM ($CDCl_3$, 100 MHz).

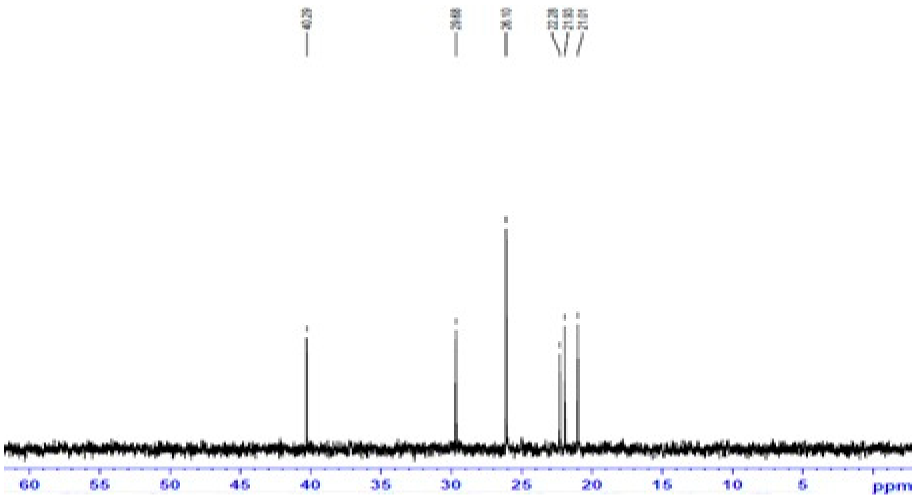

Figure S2.4.2. ^{13}C-NMR spectrum of RTM ($CDCl_3$, 100 MHz). Expansion in the area from 0.5 - 60 ppm.

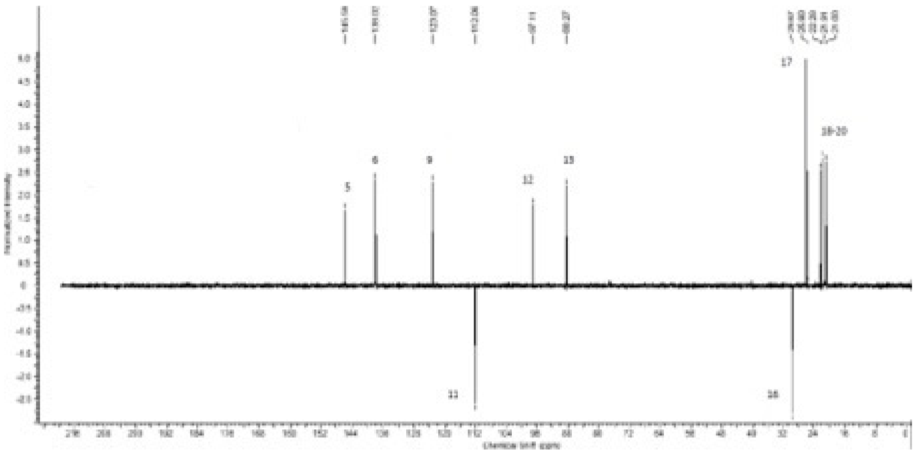

Figure S2.5. DEPT 135, ^{13}C-NMR spectrum of RTM ($CDCl_3$, 100 MHz).

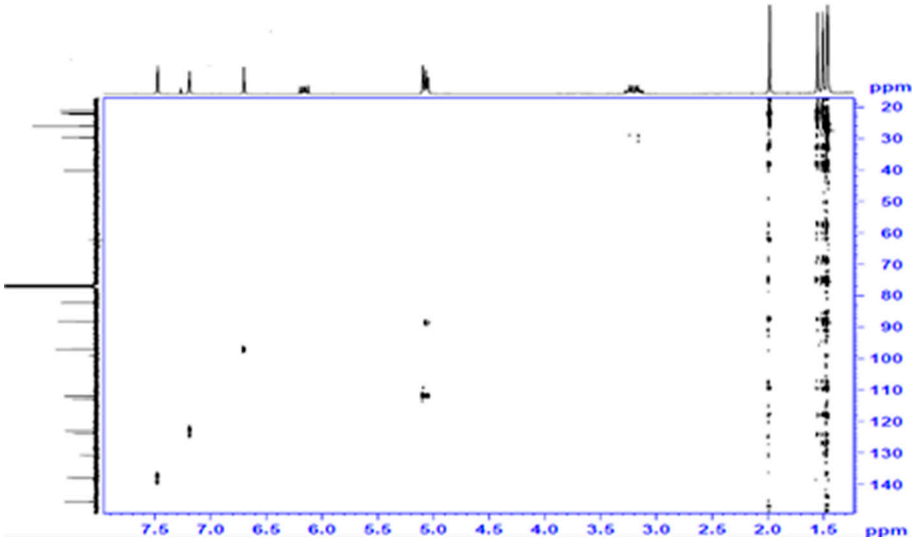

Figure S2.5.1. ^1H-^{13}C-HSQC spectrum of RTM ($CDCl_3$, 400 MHz).

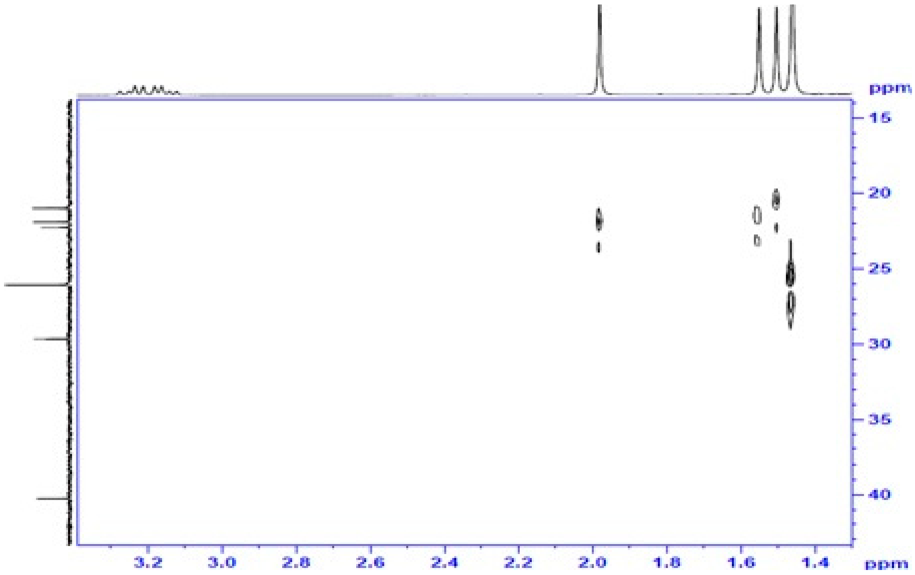

Figure S2.5.2. ^1H-^{13}C-HSQC spectrum of RTM ($CDCl_3$, 400 MHz). Expansion of the area ^1H: 1.3 - 3.4 ppm, ^{13}C: 15 - 45 ppm.

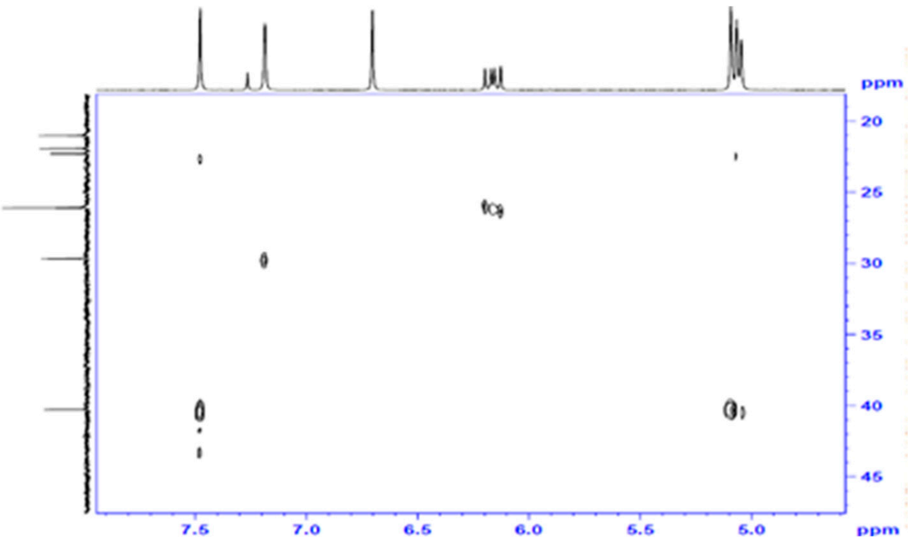

Figure S2.5.3. ^{1}H-^{13}C-HSQC spectrum of RTM ($CDCl_3$, 400 MHz). Expansion of the area ^{1}H: 4.6 - 8.0 ppm, ^{13}C: 20 – 47 ppm.

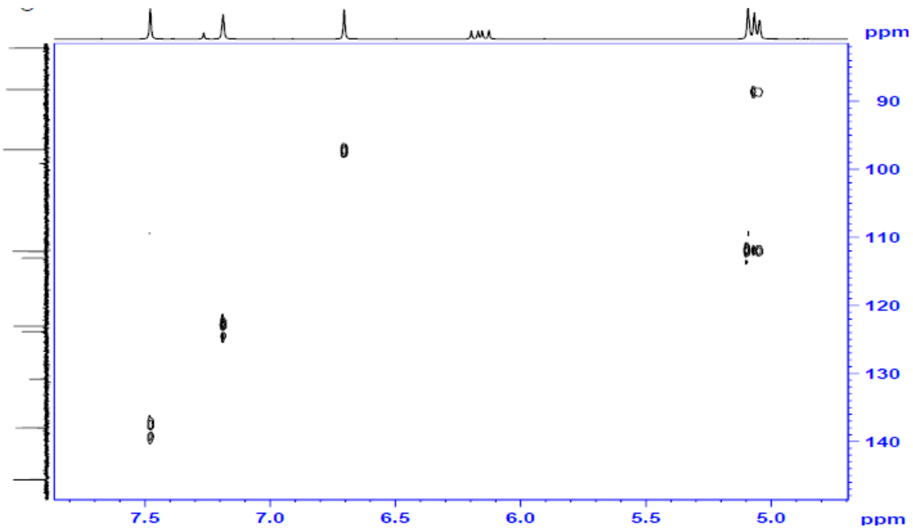

Figure S2.5.4. ^{1}H-^{13}C-HSQC spectrum of RTM ($CDCl_3$, 400 MHz). Expansion of the area ^{1}H: 4.6 - 8.0 ppm, ^{13}C: 80 - 150 ppm.

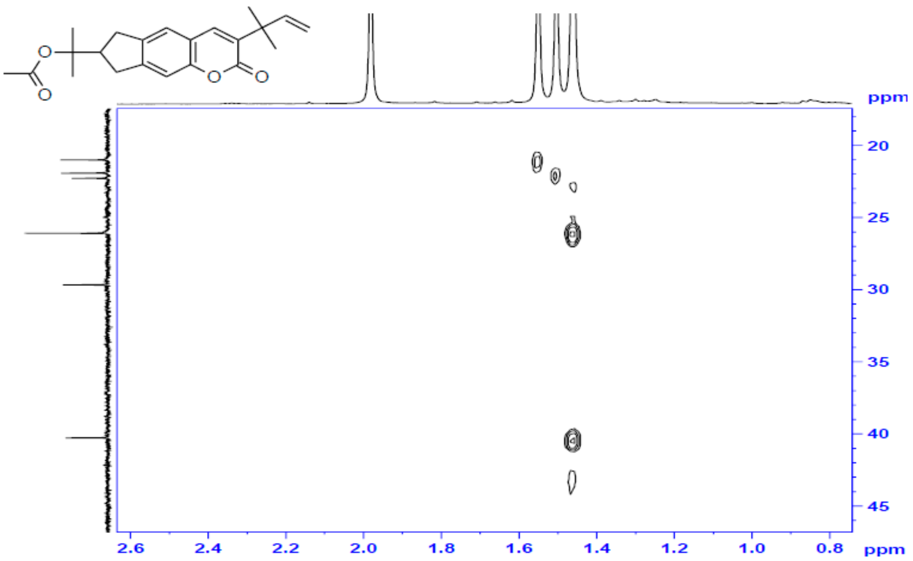

Figure S2.6.1. ^{1}H-^{13}C-HMBC spectrum of RTM ($CDCl_3$, 400 MHz). Expansion of the area ^{1}H: 0.8 - 2.6 ppm, ^{13}C: 18 - 46 ppm.

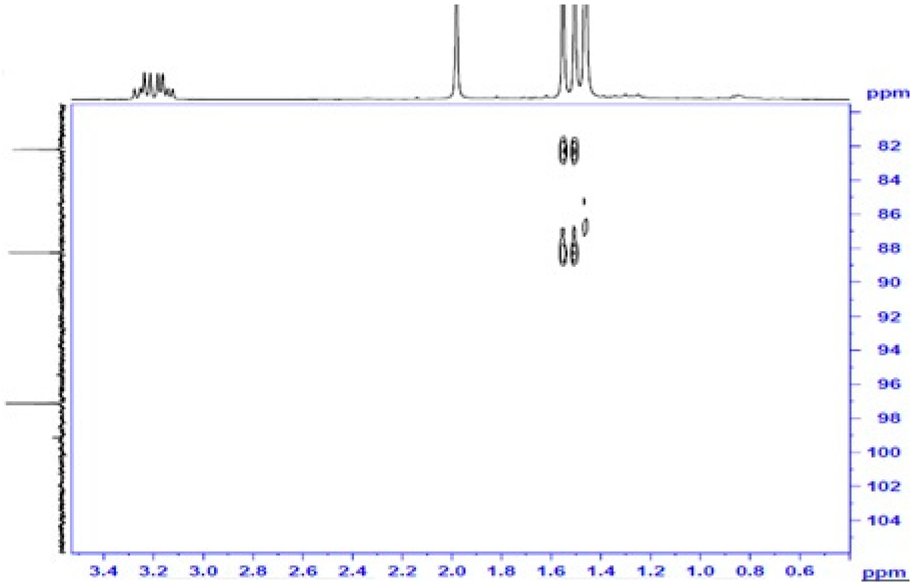

Figure S2.6.2. ^{1}H-^{13}C-HMBC spectrum of RTM ($CDCl_3$, 400 MHz). Expansion of the area ^{1}H: 0.4 - 3.5 ppm, ^{13}C: 80 - 106 ppm.

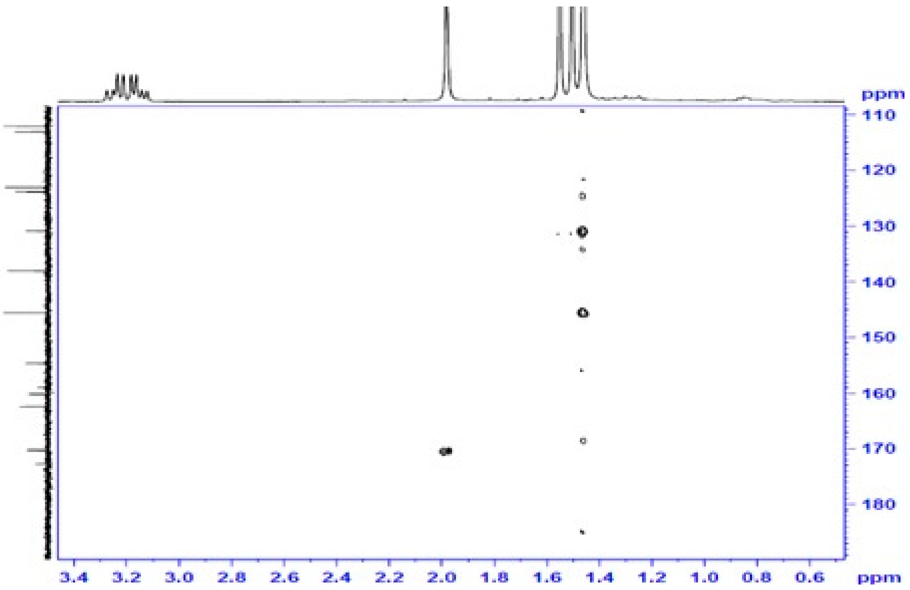

Figure S2.6.3. ^{1}H-^{13}C-HMBC spectrum of RTM ($CDCl_3$, 400 MHz). Expansion of the area ^{1}H: 0.4 - 3.4 ppm, ^{13}C: 109 - 190 ppm.

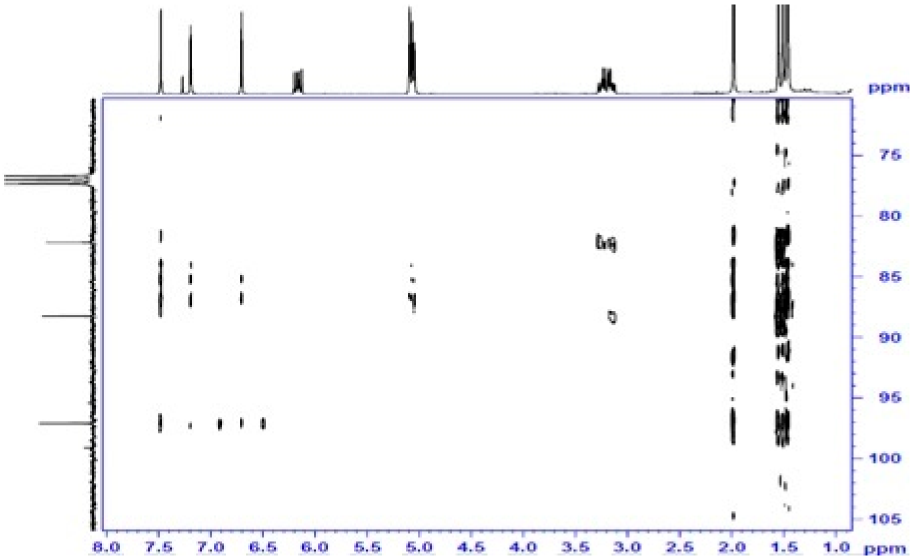

Figure S2.6.4. ^{1}H-^{13}C-HMBC spectrum of RTM ($CDCl_3$, 400 MHz). Expansion of the area ^{1}H: 0.8 – 8.0 ppm, ^{13}C: 70 - 106 ppm.

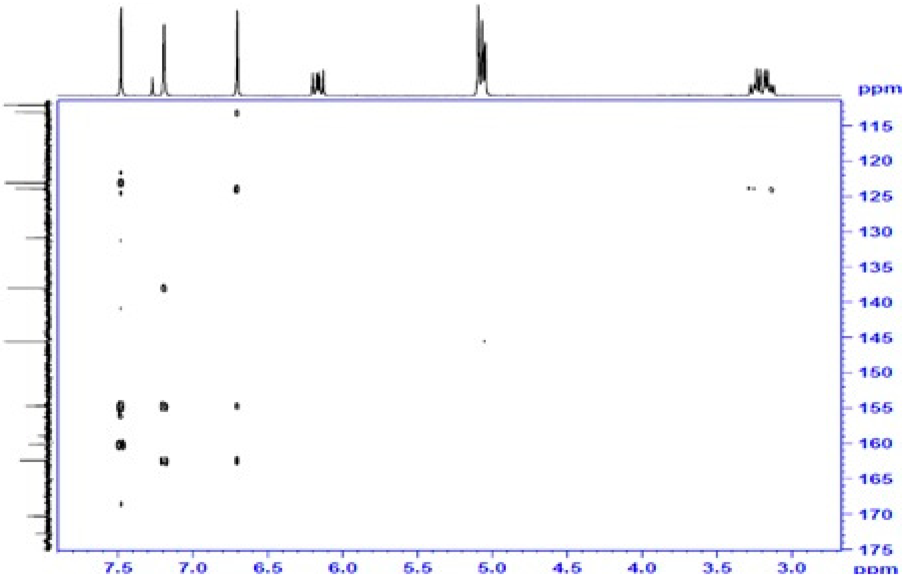

Figure S2.6.5. ^{1}H-^{13}C-HMBC spectrum of RTM ($CDCl_3$, 400 MHz). Expansion of the area ^{1}H: 2.5 – 8.0 ppm, ^{13}C: 110 - 175 ppm.

Graveolin (GRV)

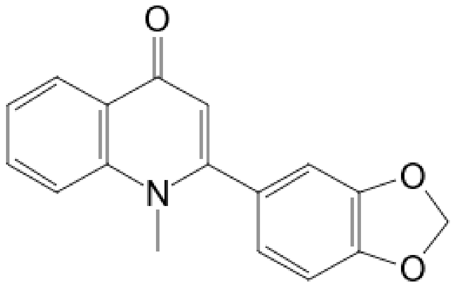

Figure S3.1. Structure of graveoline (GRV). PubChem CID = 353825 (https://pubchem.ncbi.nlm.nih.gov/compound/353825).

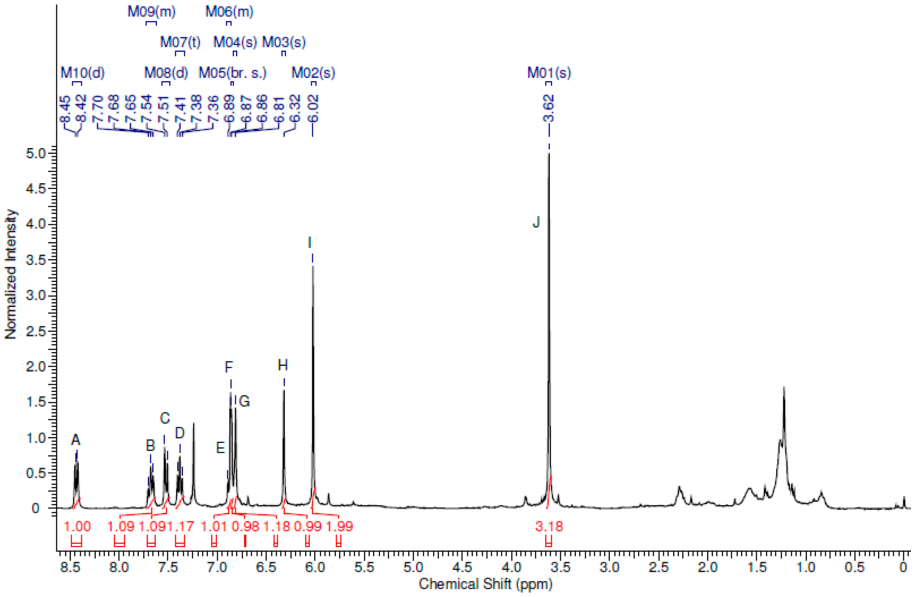

Figure S3.2.1. ^{1}H-NMR spectrum of GRV ($CDCl_3$, 400 MHz).

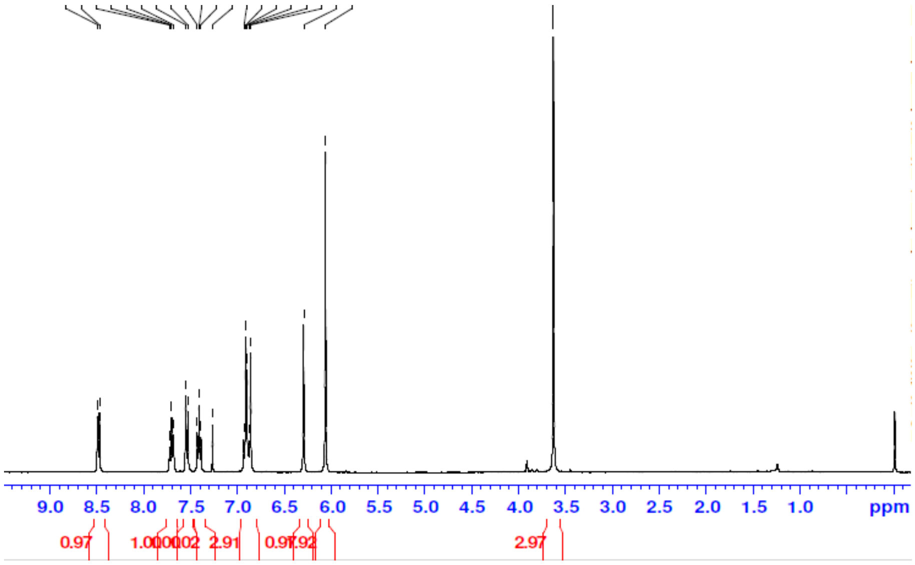

Figure S3.2.2. ^{1}H-NMR general spectrum of GRV ($CDCl_3$, 400 MHz).

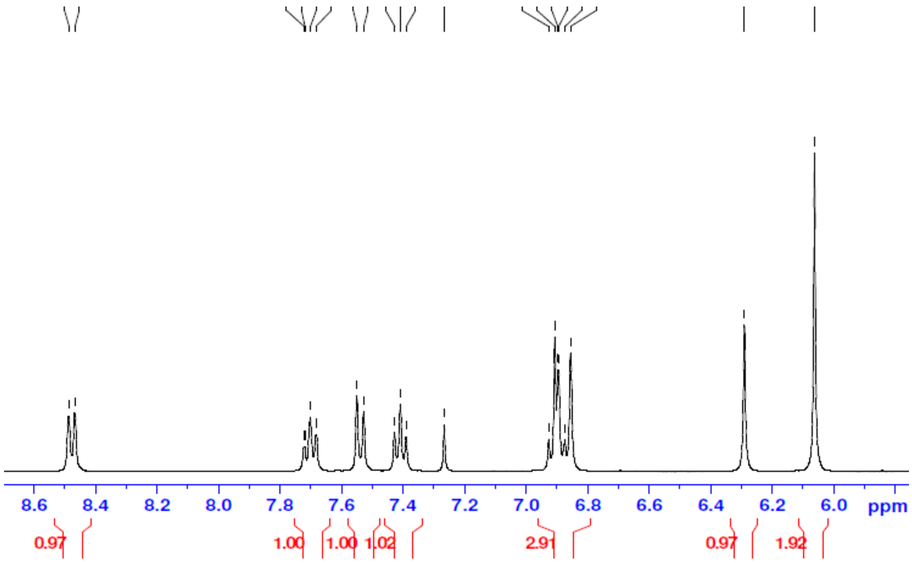

Figure S3.2.3. ^{1}H-NMR spectrum of GRV ($CDCl_3$, 400 MHz). Expansion of the area ^{1}H: 5.7 – 8.7 ppm.

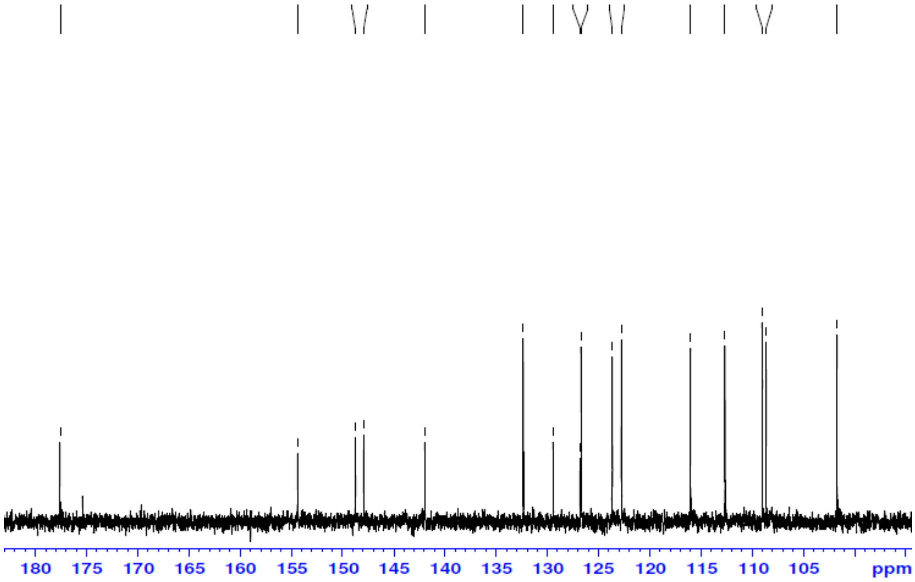

Figure S3.2.4. ^{1}H-NMR spectrum of GRV ($CDCl_3$, 100 MHz). Expansion of the area ^{1}H: 94 – 183 ppm.

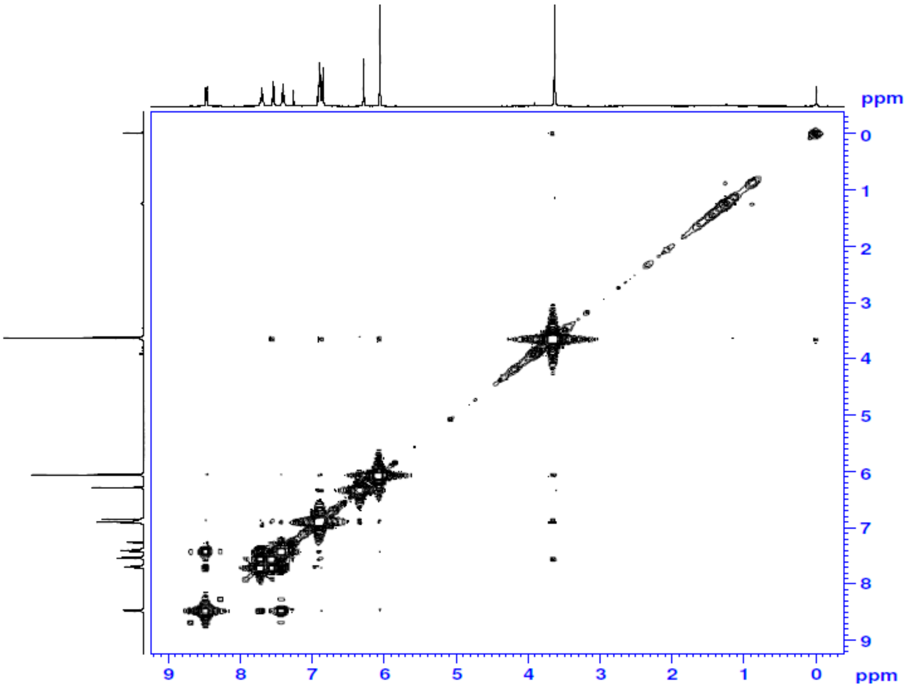

Figure S3.3.1. ^{1}H-^{1}H COSY general spectrum of GRV ($CDCl_3$, 400 MHz).

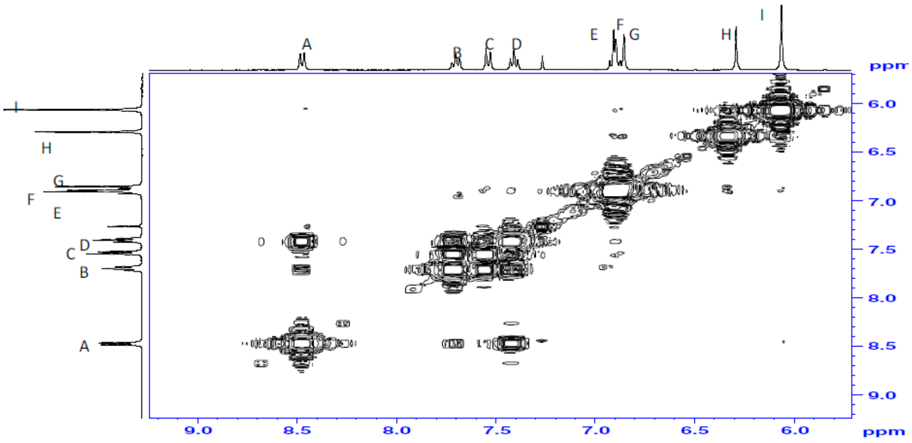

Figure S3.3.2. ^{1}H-^{1}H COSY spectrum of GRV ($CDCl_3$, 400 MHz). Expansion of the area ^{1}H: 5.7 – 9.2 ppm, ^{1}H: 5.7 – 9.2.

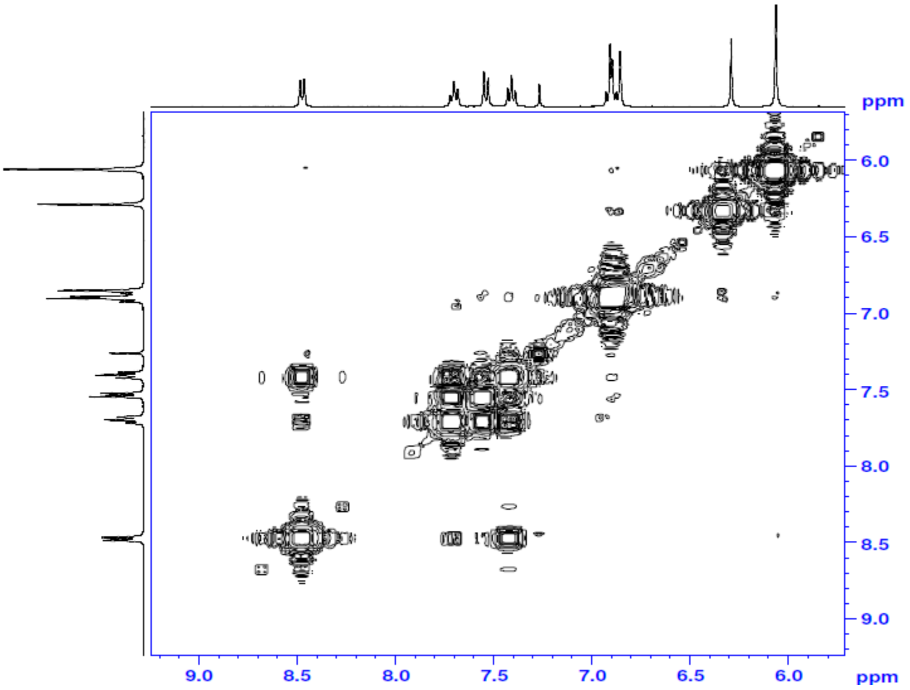

Figure S3.3.3. ^1H-^1H COSY spectrum of GRV ($CDCl_3$, 400 MHz). Expansion of the area ^1H: 5.7 – 9.2 ppm, ^1H: 5.7 – 9.2.

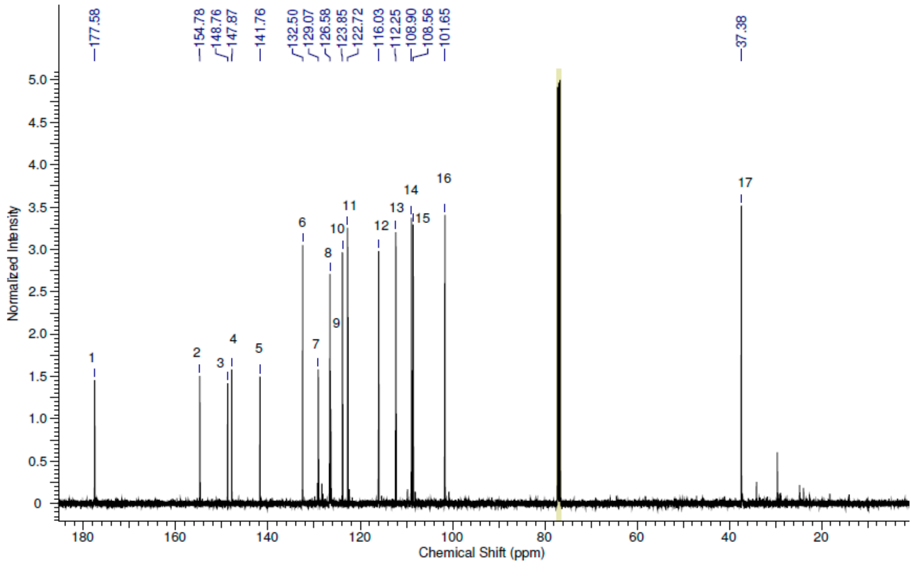

Figure S3.4.1. ^{13}C-NMR spectrum of GRV ($CDCl_3$, 100 MHz).

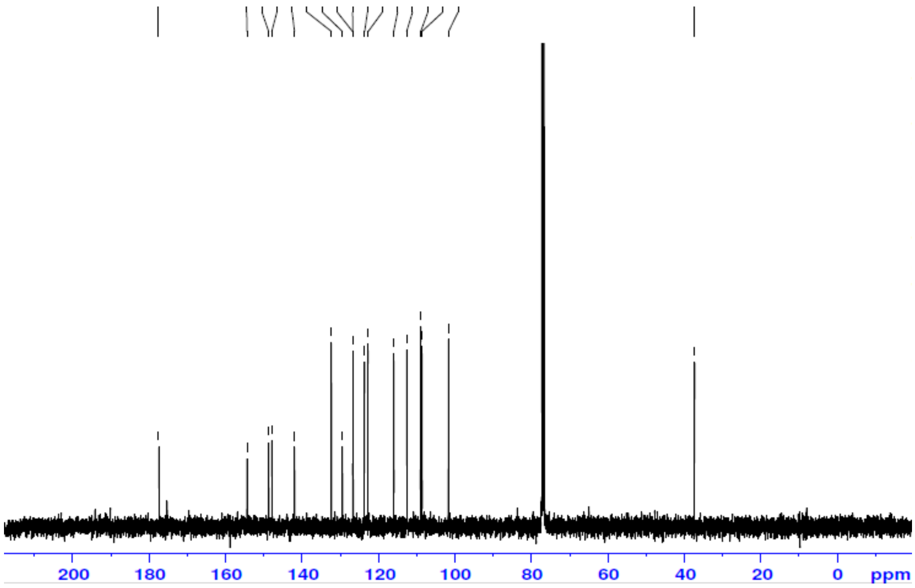

Figure S3.4.2. ^{13}C-NMR spectrum of GRV ($CDCl_3$, 100 MHz).

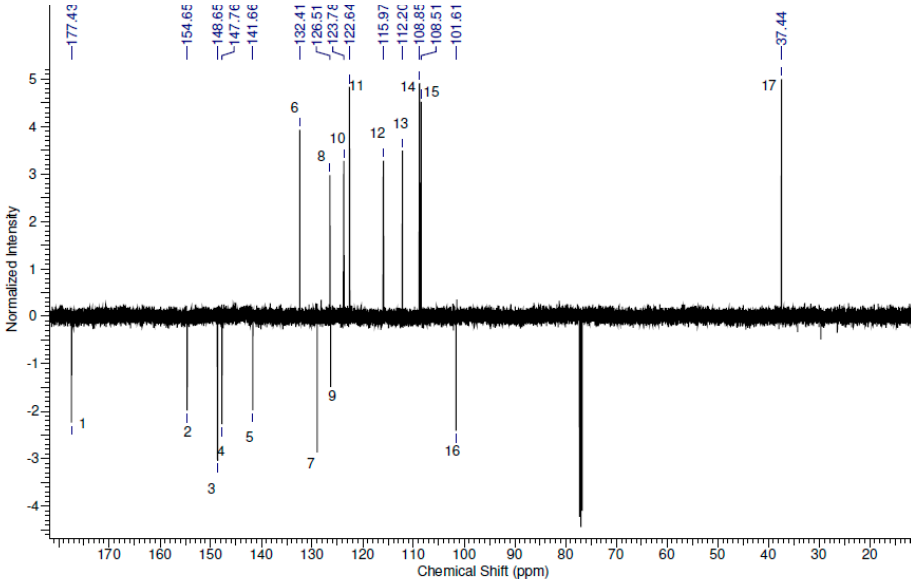

Figure S3.5. APT, ^{13}C-NMR spectrum of GRV ($CDCl_3$, 400 MHz).

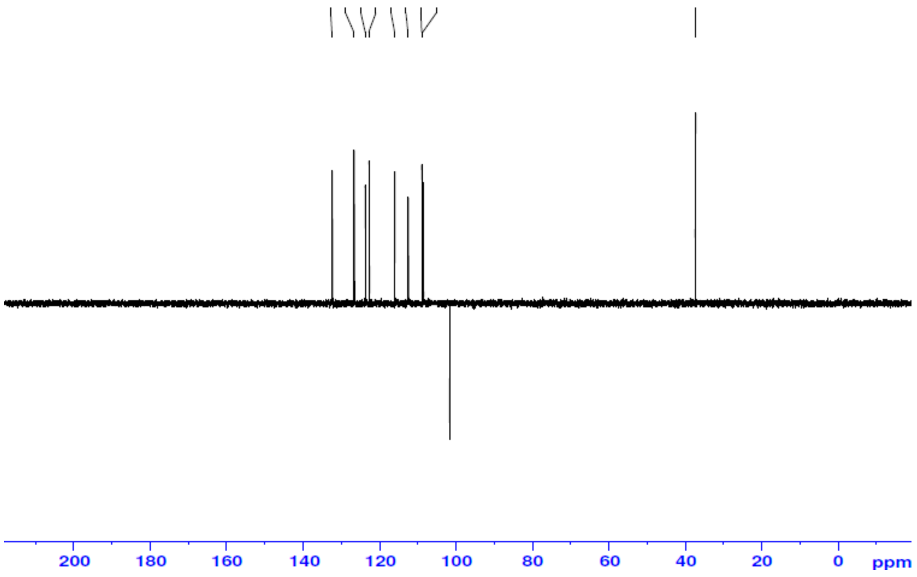

Figure S3.6. DEPT 135, ^{13}C-NMR spectrum of GRV ($CDCl_3$, 100 MHz).

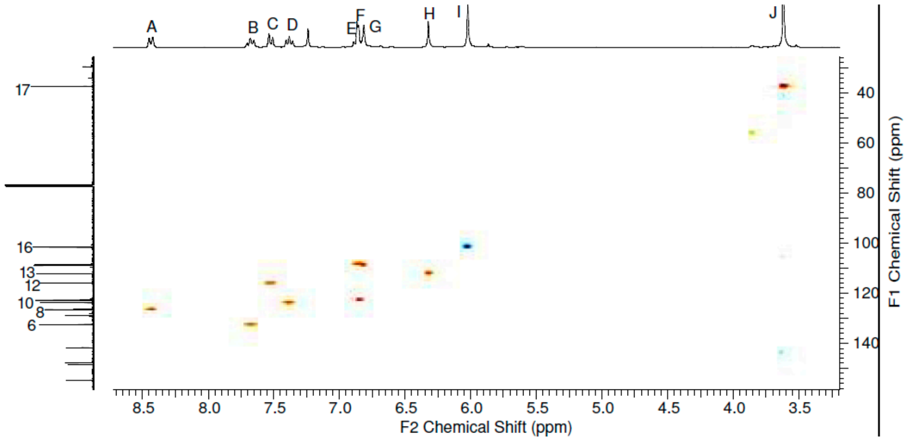

Figure S3.7.1. ^{1}H-^{13}C-HSQC spectrum of GRV ($CDCl_3$, 400 MHz).

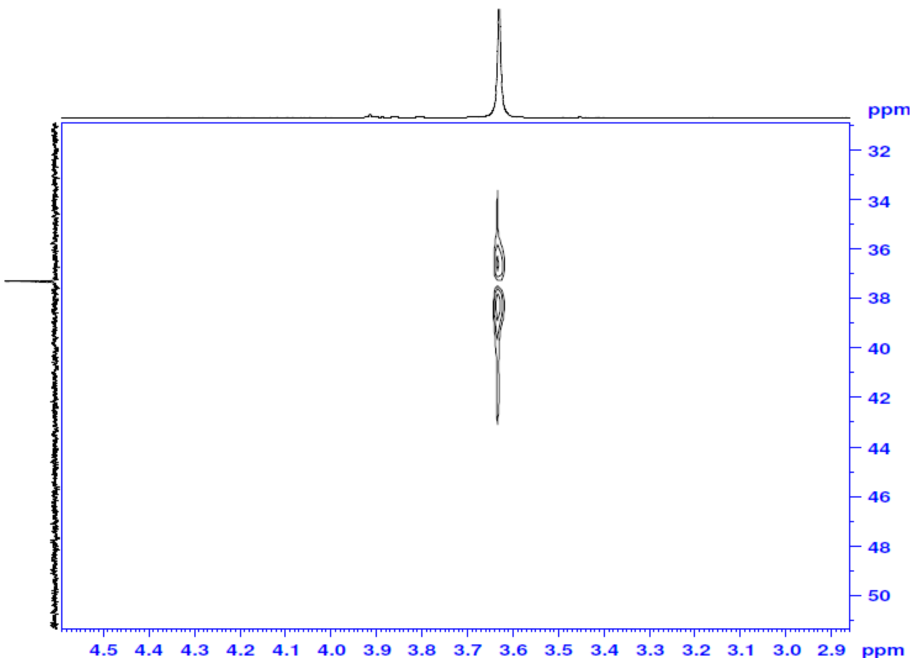

Figure S3.7.2. ^{1}H-^{13}C-HSQC spectrum of GRV ($CDCl_3$, 400 MHz). Expansion of the area ^{1}H: 2.9 - 4.6 ppm, ^{13}C: 31 - 51.

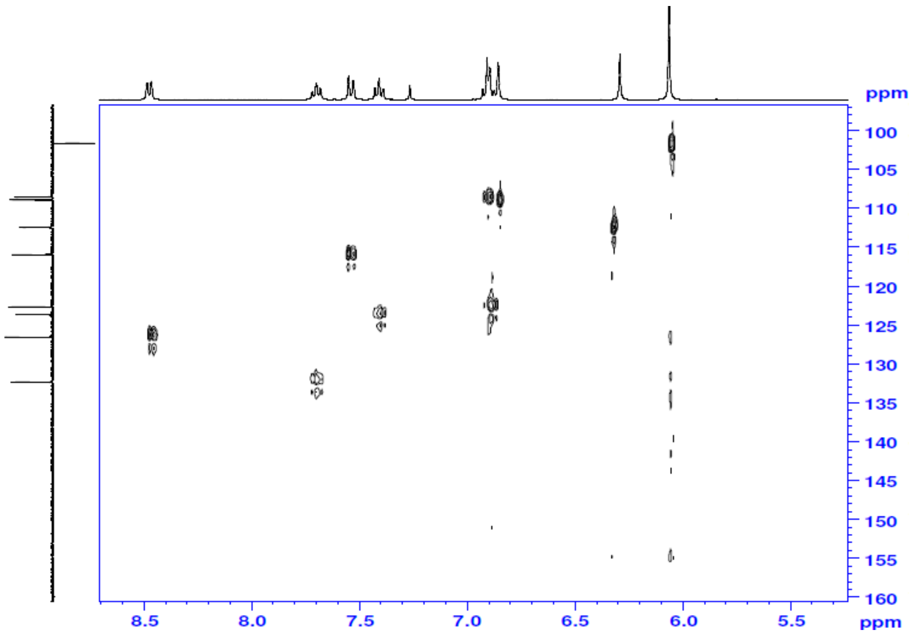

Figure S3.7.3. ^{1}H-^{13}C-HSQC spectrum of GRV ($CDCl_3$, 400 MHz). Expansion of the area ^{1}H: 5.3 - 8.7 ppm, ^{13}C: 95 - 160.

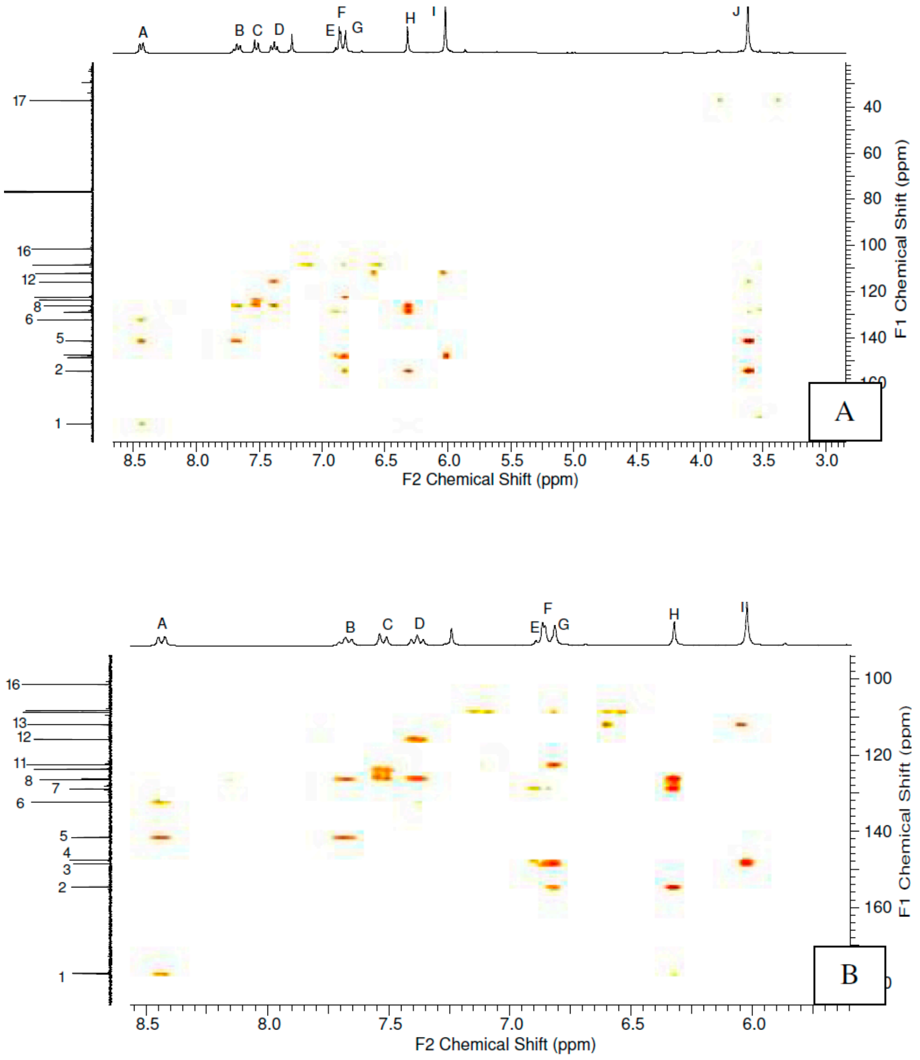

Figure S3.8.1. ^{1}H-^{13}C-HMBC spectrum of GRV ($CDCl_3$, 400 MHz). A: General spectrum.; B: Expansion of the area ^{1}H: 5.0 - 8.8 ppm, ^{13}C: 90 - 185 ppm.

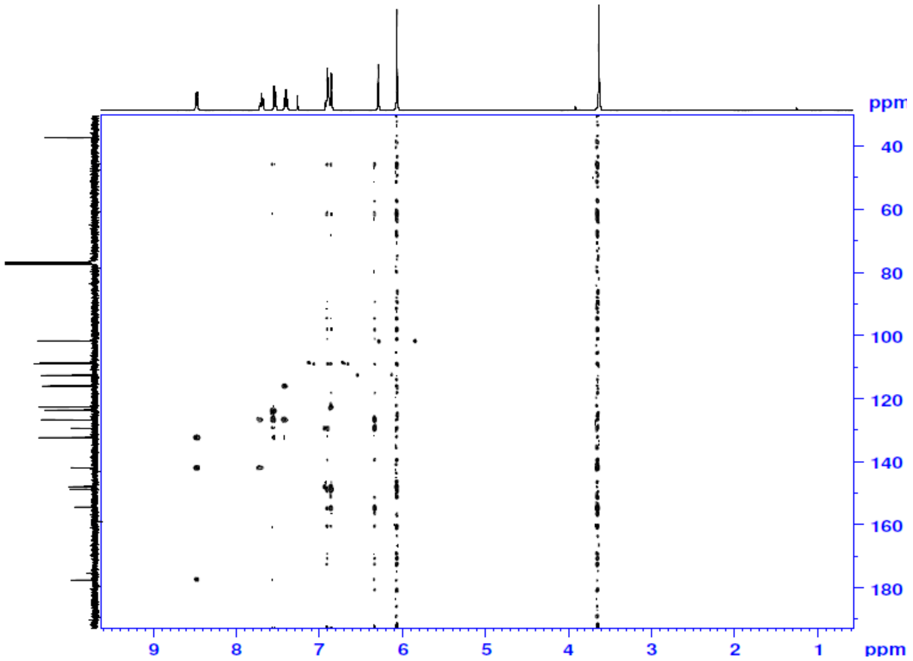

Figure S3.8.2. ^{1}H-^{13}C-HMBC general spectrum of GRV ($CDCl_3$, 400 MHz).

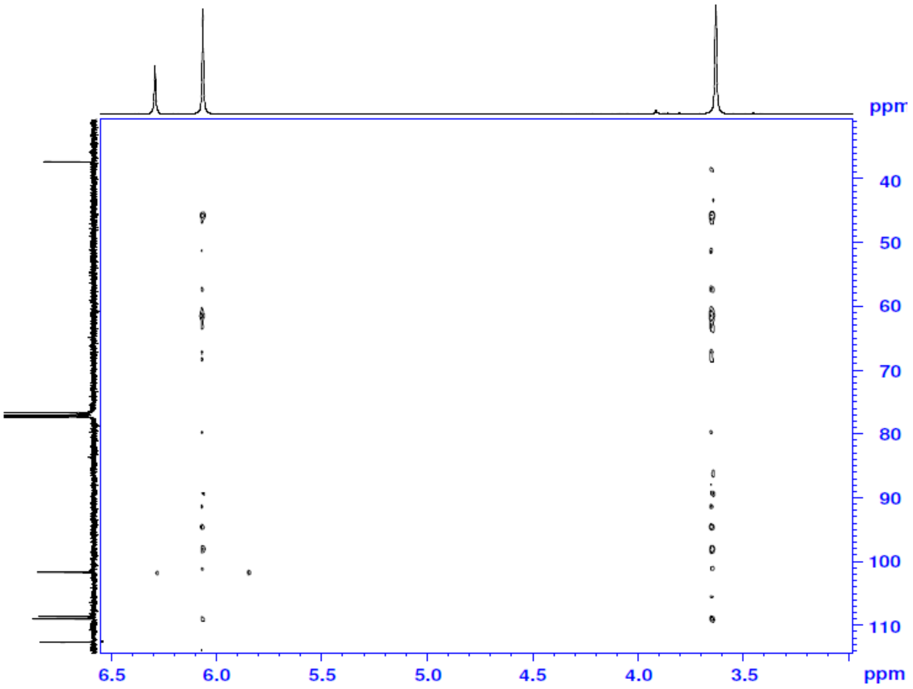

Figure S3.8.3. ^{1}H-^{13}C-HMBC spectrum of GRV ($CDCl_3$, 400 MHz). Expansion of the area ^{1}H: 3.0 - 6.6 ppm, ^{13}C: 30 - 115 ppm.

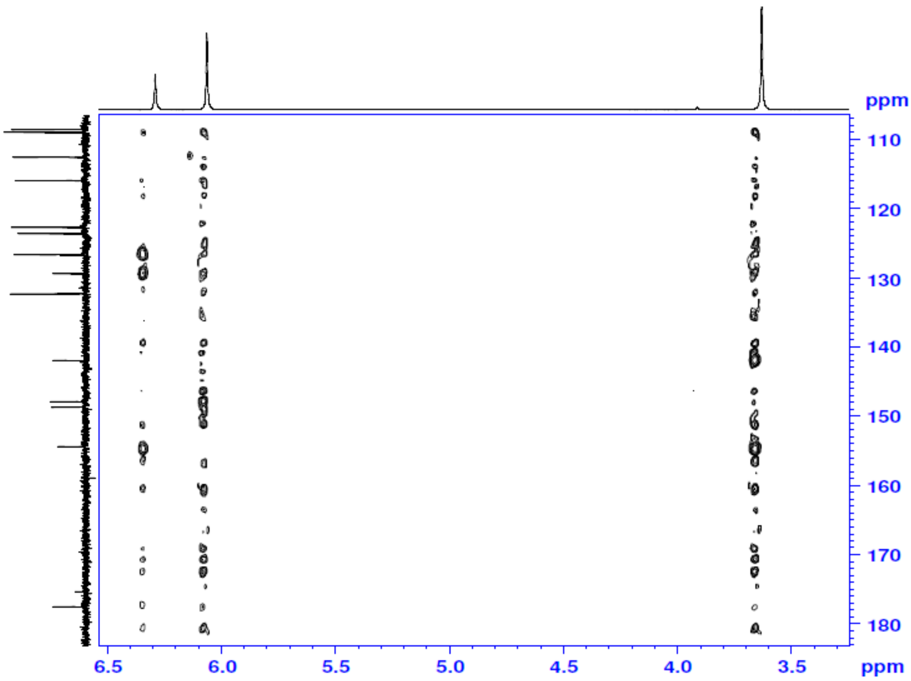

Figure S3.8.4. ^1H-^{13}C-HMBC spectrum of GRV ($CDCl_3$, 400 MHz). Expansion of the area ^1H: 3.0 - 6.6 ppm, ^{13}C: 107 - 183 ppm.

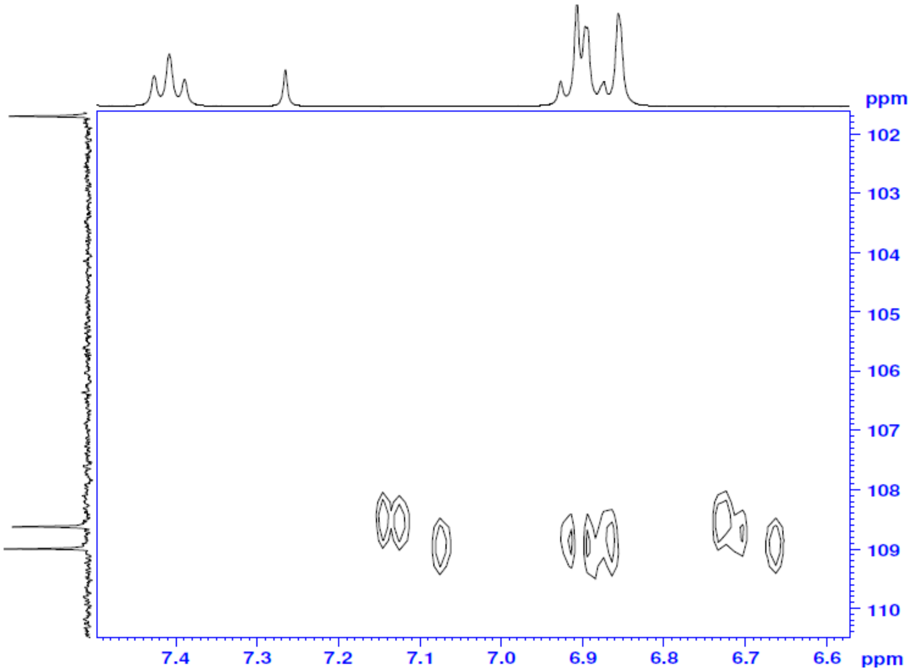

Figure S3.8.5. ^1H-^{13}C-HMBC spectrum of GRV ($CDCl_3$, 400 MHz). Expansion of the area ^1H: 6.4 - 7.5 ppm, ^{13}C: 99 - 115 ppm.

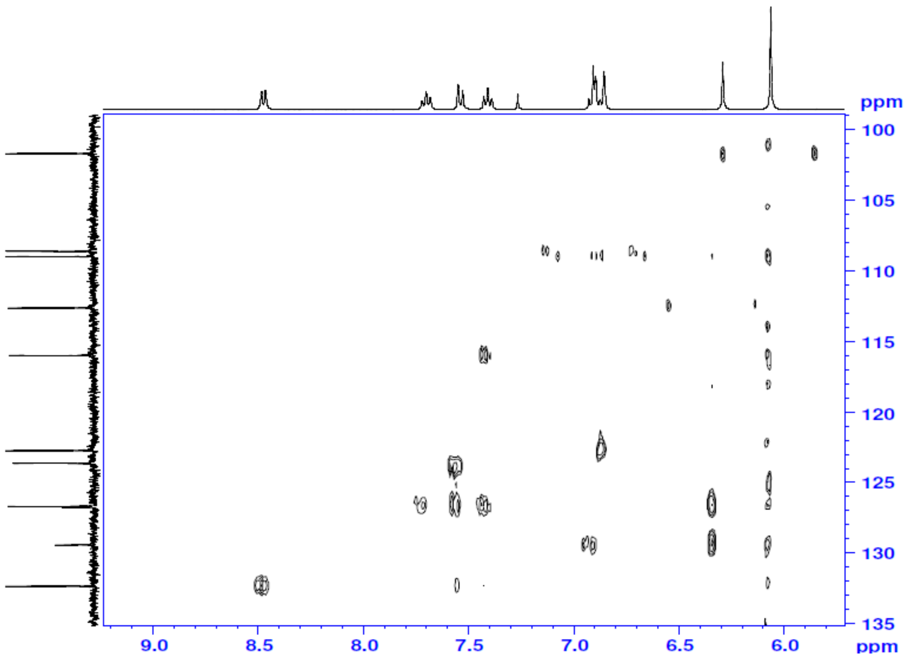

Figure S3.8.6. ^{1}H-^{13}C-HMBC spectrum of GRV ($CDCl_3$, 400 MHz). Expansion of the area ^{1}H: 5.7 – 9.2 ppm, ^{13}C: 99 - 135 ppm.

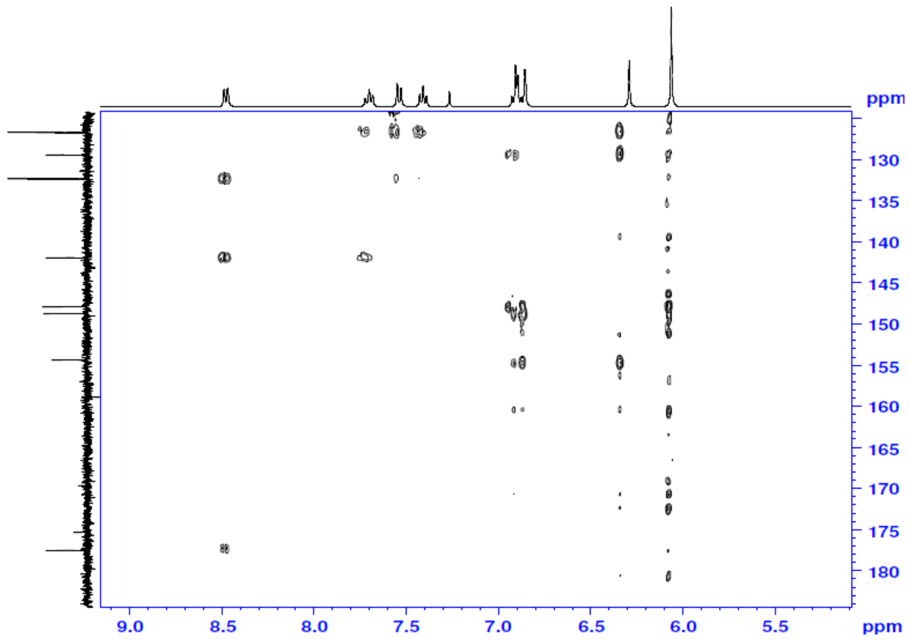

Figure S3.8.7. ^{1}H-^{13}C-HMBC spectrum of GRV ($CDCl_3$, 400 MHz). Expansion of the area ^{1}H: 5.1 – 9.1 ppm, ^{13}C: 124 - 185 ppm.

Data Availability Statement: The datasets generated or analyzed during the present study are available from the corresponding authors.

Conflicts of Interest: The authors declare no conflict of interest.

References

1. Quintanilla-Licea, R.; Mata-Cárdenas, B.D.; Vargas-Villarreal, J.; Bazaldúa-Rodríguez, A.F.; Ángeles-Hernández, I.K.; Garza-González, J.N.; Hernández-García, M.E. Antiprotozoal activity against *Entamoeba histolytica* of plants used in northeast mexican traditional medicine. Bioactive compounds from *Lippia graveolens* and *Ruta chalepensis*. *Molecules* **2014**, *19*, 21044–21065, doi:10.3390/molecules191221044.
2. Bazaldúa-Rodríguez, A.F.; Quintanilla-Licea, R.; Verde-Star, M.J.; Hernández-García, M.E.; Vargas-Villarreal, J.; Garza-González, J.N. Furanocoumarins from *Ruta chalepensis* with amebicide activity. *Molecules* **2021**, *26*, 1–11, doi:10.3390/molecules26123684.
3. Quintanilla-Licea, R.; Mata-Cárdenas, B.; Vargas-Villarreal, J.; Bazaldúa-Rodríguez, A.; Verde-Star, M. Antiprotozoal activity against *Entamoeba histolytica* of furocoumarins isolated from *Ruta chalepensis*. *Planta Med.* **2016**, *81*, S1–S381, doi:10.1055/s-0036-1596528.
